# Comparing nutrient reference concentrations in Nordic countries with focus on lowland rivers

**DOI:** 10.1007/s13280-020-01370-4

**Published:** 2020-09-15

**Authors:** Eva Skarbøvik, Jukka Aroviita, Jens Fölster, Anne Lyche Solheim, Katarina Kyllmar, Katri Rankinen, Brian Kronvang

**Affiliations:** 1 Norwegian Institute for Bioeconomy Research, NIBIO, P.O. Box. 115, 1431 Ås, Norway; 2 Freshwater Centre, Finnish Environment Institute, PO Box 413, 90014 Oulu, Finland; 3 Department of Aquatic Sciences and Assessment, Swedish University of Agricultural Sciences, PO Box 7050, 750 07 Uppsala, Sweden; 4 Norwegian Institute of Water Research, NIVA, Gaustadalléen 21, 0349 Oslo, Norway; 5 Department of Soil and Environment, Swedish University of Agricultural Sciences, PO Box 7014, 750 07 Uppsala, Sweden; 6 Biodiversity Centre, Finnish Environment Institute, Latokartanonkaari 11, 00790 Helsinki, Finland; 7 Department of Bioscience, Aarhus University, Vejlsøvej 25, 8600 Silkeborg, Denmark

**Keywords:** Nordic, Nutrients, Reference conditions, Rivers, WFD

## Abstract

Reference conditions of water bodies are defined as the natural or minimal anthropogenically disturbed state. We compared the methods for determining total phosphorus and total nitrogen concentrations in rivers in Finland, Norway and Sweden as well as the established reference conditions and evaluated the possibility for transfer and harmonisation of methods. We found that both methods and values differed, especially for lowland rivers with a high proportion of agriculture in the catchment. Since Denmark has not yet set reference conditions for rivers, two of the Nordic methods were tested for Danish conditions. We conclude that some of the established methods are promising but that further development is required. We moreover argue that harmonisation of reference conditions is needed to obtain common benchmarks for assessing the impacts of current and future land use changes on water quality.

**Electronic supplementary material:**

The online version of this article (10.1007/s13280-020-01370-4) contains supplementary material, which is available to authorised users.

## Introduction

Reference conditions (RC) represent a baseline for assessing the current ecological status of water bodies and can be quantified by, for instance, biological indicators and nutrient concentrations. The EU Water Framework Directive (WFD; EC 2000) defines RC as “no, or only very minor, anthropogenic alterations (…) for the surface water body types from those normally associated with that type under undisturbed conditions”. This definition leaves space for interpretation, especially in terms of the “very minor” deviation from undisturbed conditions (CIS Guidance 2003a). Stoddard et al. (2006) advocated that RCs in rivers should reflect *minimally disturbed conditions* or ‘the condition of streams in the absence of significant human disturbance’, but in lowland rivers such conditions are rarely found. In consequence, several methods have been advocated for establishing nutrient RCs (CIS Guidance 2003a; Stoddard et al. 2006; Poikane et al. 2019). In the presence of pristine water bodies, the preferred method to determine nutrient RCs is use of monitoring data on nutrients and nutrient-sensitive biological indicators. In the absence of pristine water bodies, a variety of other methods can be used, including models, data and information derived from historical records, expert judgement or a combination of these.

**Fig. 1:**
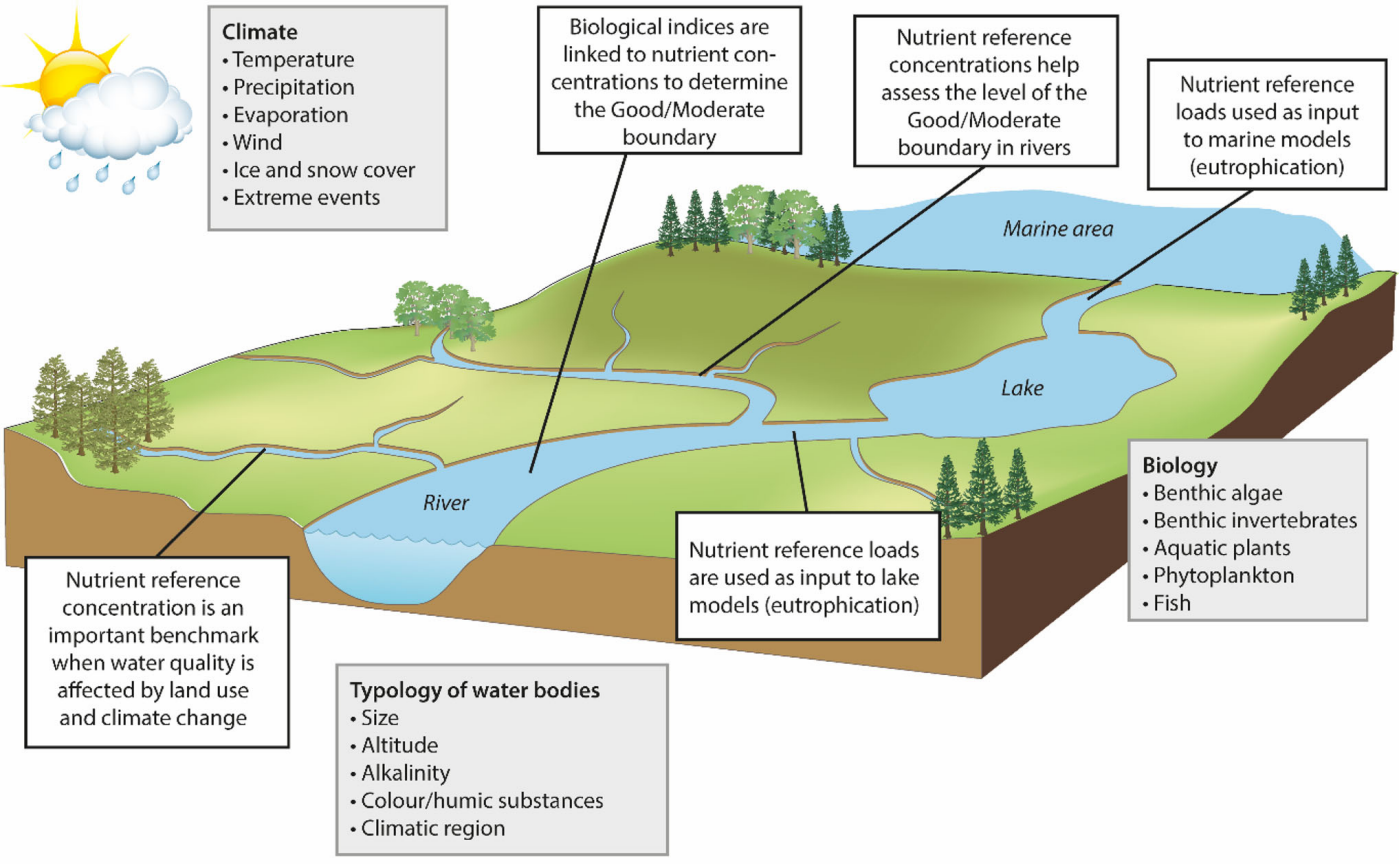
Purposes of reference conditions illustrated in a river basin

Determination of RCs is important since the RC concept serves different purposes (Stoddard et al. 2006; Carvalho et al. 2019; Fig. 1). Hawkins et al. (2010) noted that most of the published studies on ecological assessments during the last 25 years have depended on the determination of an “ecological benchmark for context”. In the WFD, RC is also used as a basis for establishing the boundary between “good” and “moderate” ecological status (G/M boundary). This can be done either directly, for example, by multiplying the nutrient RC with a constant or by evaluating whether a suggested G/M boundary, which is often established from dose–response relationships using nutrient-sensitive biology, is within a reasonable distance from the RC, i.e. showing only “slight” deviation from RC (Bald et al. 2005; Carvalho et al. 2008, Huser and Fölster 2013, Lyche Solheim et al. 2008a, 2019). Biological data are often used to link tolerance levels for biological quality elements to nutrients or related stressors, such as the Saprobic Diatom Index for diatoms (Rott et al. 2003), the Periphyton Index for trophic status for non-diatom algae (Schneider and Lindstrøm 2011), the Trophic Diatom Index for phytobenthos (Kelly and Whitton 1995) and the ASPT index for benthic invertebrates (Armitage et al. 1983). Moreover, riverine nutrient reference concentrations can be transferred to reference loads and serve as input data to lake models (Poikane et al. 2010) or marine models (Erichsen et al. 2017). When water bodies fail to achieve good status, it is necessary to implement mitigation measures and since these often are costly, determination of nutrient RCs and the critical G/M boundary are important also from an economic point of view (Phillips et al. 2018).

Nutrient RCs are relevant in yet another important context, namely as a benchmark for water quality during the coming land use changes caused by the expected transition to a bioeconomy (Hertel et al. 2012; O’Brien et al. 2017). This change may increase the need for biomass for various purposes, among others as a substitution for fossil fuels, which again may result in intensification of both forestry and agriculture (for a more detailed discussion of this topic, see Rakovic et al. (this issue) and Marttila et al (this issue)). A related concern is that such land use changes may negatively affect freshwater quality and quantity (Ahtiainen and Huttunen 1999; Rosegrant et al. 2012). Harmonisation of Nordic methods, including nationally established RCs for nutrients, will be required to compare impacts of the land use changes across countries. As noted by Poikane et al. (2019), the G/M boundaries for European rivers vary widely among the EU countries, implying variations in the associated RC levels as well.

In this paper, we explored the established reference levels for the nutrients total phosphorus (TP) and total nitrogen (TN) in three Nordic countries (Finland, Norway and Sweden) as well as the methods used for their determination. We also assessed to which degree the methods could be transferred to Denmark where national RCs for nutrients in rivers have not yet been established.

## Methodology

We collected all available literature on the implementation of the WFD in the Nordic countries, including national guidelines, underlying reports and relevant international literature.

For river typology, we used the work of the so-called Geographical Intercalibration Groups (GIGs) that were established during the WFD Common Implementation Strategy (CIS) process to handle the intercalibration of the High/Good and Good/Moderate class boundaries for each biological quality element in each common intercalibration type (van de Bund 2009). Common criteria for water body types are based on geographical region, catchment size, altitude, alkalinity and organic matter (EC 2018). Most of Sweden and all of Finland and Norway belong to the “Northern GIG” and share five river types in lowland and mid-altitude regions. High-altitude rivers are not discussed in this paper since ample pristine water bodies exist within this category, and they are less likely to be impacted by the land use changes occurring due to the shift to bioeconomy. Denmark and the southernmost parts of Sweden are located within the Central/Baltic geographical region of Europe (“CB-GIG”) and have two lowland river types in common. To these seven river types, we added one more common type—rivers draining clay-rich soils.

In Finland and Norway, RCs for nutrients in different river types are available in the most updated national guidelines (Direktoratsgruppen 2018; Aroviita et al. 2019). In Sweden, the national methodology is based on site-specific predictive modelling, and calculation of ranges of reference values for each of the river types chosen for this study was therefore required.

In Denmark, RCs have not yet been set for rivers, and we therefore compared the following three methods to propose possible RC values for Danish rivers:

1. Direct use of water quality data from a monitoring programme applied in smaller Danish catchments with less than 10% agriculture (Kronvang et al. 2015). The conditions in these are comparable those defined by Stoddard et al. (2006) as “least disturbed conditions”, i.e. the best available habitat conditions given today’s state of the landscape. The streams were monitored for the first time in 2004/2005, and of these 16 were included in the Danish National Environmental and Nature Monitoring Programme and have since 2011 been monitored every 3rd year for daily discharge and monthly water chemistry parameters, including nutrient concentrations (map shown in Fig. 4c; supplementary material).
2. Modelling based on the Swedish model (see details in the below section on national methods). Two model versions, one without and one with agricultural impacts (Eqs. S3 and S4 in the supplementary material), were applied to the 16 rivers under (1).
3. Modelling based on the Norwegian model for rivers in clay-rich soils (see details in the below section on national methods). Since none of the small monitored streams were located in clay-rich soils, the Norwegian model was tested on the Uggerby River in northern Jutland where Quaternary marine clay soils underlie the aeolian sandy/sandy loam soils (map in Fig. 4d).

## Definitions of reference criteria for selection of near-pristine reference sites in the Nordic countries

The first step in the process of determining reference conditions is to identify true reference sites having near-pristine conditions. Where Denmark has not yet defined criteria for rivers, Finland and Norway have defined relatively similar reference criteria (Table 1). Sweden has divided the criteria definition into two categories depending on the amount of agriculture in the catchment. Thus, for rivers with more than 10% agriculture in the catchment, RC is defined as the runoff from tile-drained fallow land. This differs from Norway and Finland where RCs also in agricultural lowland rivers mainly are based on rivers draining unmanaged forests, peatlands or moorland.

**Table 1:** National reference criteria for selecting reference sites in rivers in the four Nordic countries studied (van de Bund 2009; HaV 2017; Aroviita et al. 2019)

Country	Definition	Criteria
Denmark	Not yet defined on a national level	
Finland	High status systems with minimal anthropogenic pressure	< 10% agriculture < 5% forestry < 0.8% urban land
Norway	Waterbodies with little or no anthropogenic pressure	< 10% agriculture Population density < 5 pe/km^2^ No point source pollution*
Sweden	In rivers with < 10% agricultural soils	< 25 µg/l of Tot-P
	In rivers with > 10% agricultural soils	Unfertilised fallow on tile-drained land**

*These criteria were used to validate reference sites in the intercalibration of the reference values and high/good boundaries in the intercalibration process in NGIG

**Based on the modelled leaching from unfertilised fallow on tile-drained land and under various climate conditions and soil types (Johnsson et al. 2016). This means that grazing, but not fertilisation or tillage is present under reference conditions

## National methods to determine RCs

**Fig. 2:**
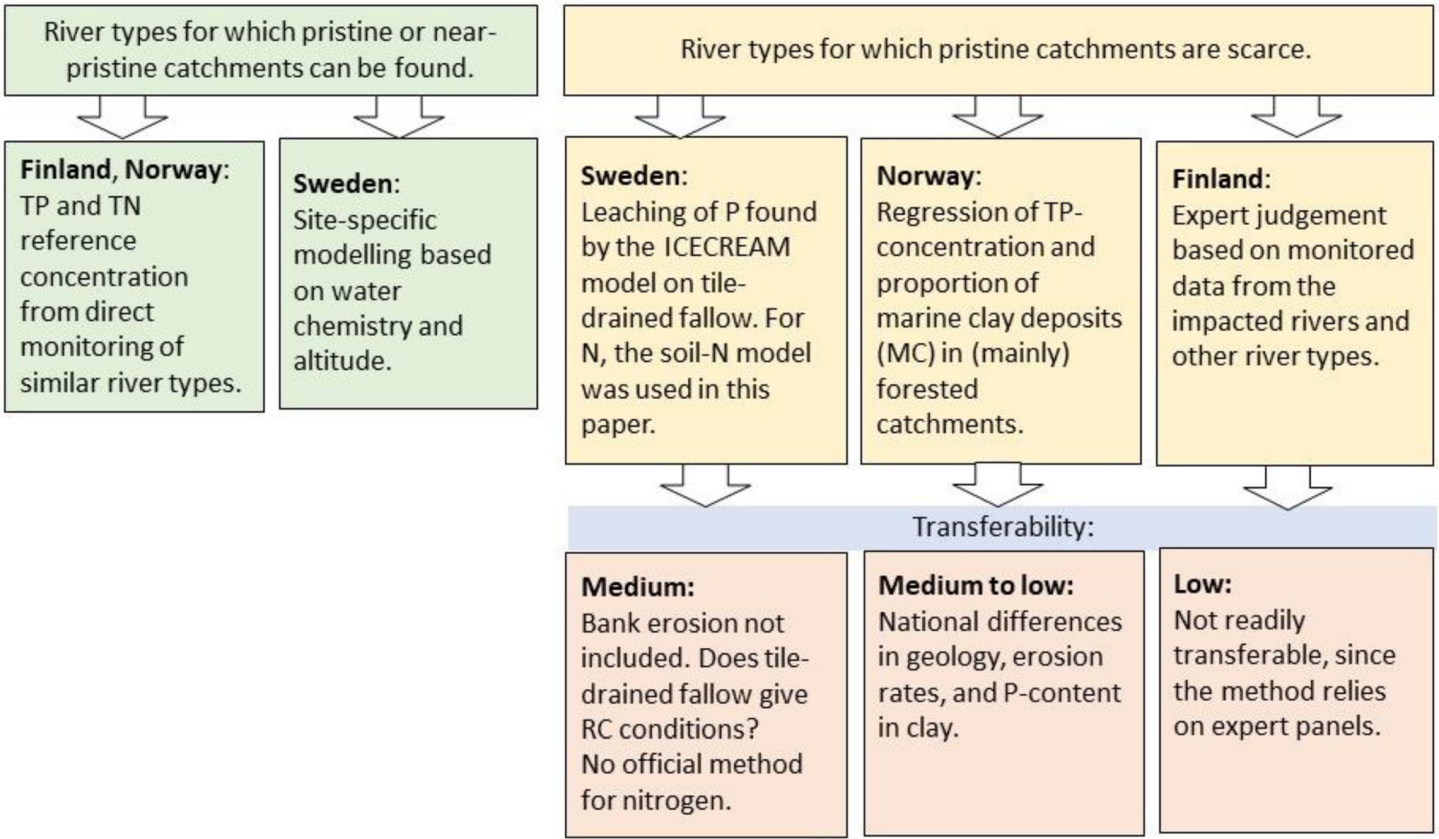
Chart of the main methods used in the countries for two sets of river types—with pristine or near-pristine conditions (left) and without pristine or near-pristine conditions (right), including assessment of transferability between countries

Figure 2 presents an overview of the national methods used in each of the three Nordic countries. The methods varied less for river types for which pristine or near-pristine conditions exist (left part of the figure) than for river types for which pristine conditions are scarce (right part of the figure).

A more thorough description of the national methods for defining reference conditions is given in the supplementary material section, both for river types with presence and absence of pristine or near-pristine catchments (principally lowland rivers with a high proportion of agricultural activity). Below, a short description is given of the methodologies used in the three countries.

In **Finland**, nutrient reference values were established based on expert judgement of monitoring data from minimally disturbed rivers of other river types and from data on disturbed rivers draining agricultural and clay-rich catchments. A national review panel evaluated the boundaries. Based on annual statistics, review panel work and tests with preliminary classification results, river type-specific High/Good status class boundaries for TP and TN were set as the 75th–90th percentiles of the nutrient concentrations among the reference or least disturbed rivers.

For **Norwegian** rivers draining clay-rich soils, a considerable part of the particulate phosphorus derives from the P in the clay-mineral apatite (Semb 1986), and the correlation in these rivers between suspended solids and TP is therefore usually good (Skarbøvik and Roseth 2014). A total of five streams draining clay-rich soils in catchments mainly covered by forest were used to produce a simple linear regression between the annual mean TP concentration and the proportion of the catchment area covered by marine clay (MC) (Lyche Solheim et al. 2008b; Eq. S1 in the supplementary material). MC was found by using superficial deposit maps without considering the depth of the clay deposits. Supposing 100% clay coverage, the maximum reference TP concentration would be 75 µg/l, but as no Norwegian rivers with catchments larger than 10 km^2^ have such a high clay coverage, the maximum RC for TP was set to 40 µg/l (Direktoratsgruppen 2018). Since nitrogen levels are not believed to be affected by the clay content, the RC for TN is the same as that for national lowland river types that are humic and have medium to high calcium contents.

**Sweden** used predictive modelling to set TP reference conditions in rivers. The model used is a linear regression of TP as a function of water colour value, non-marine Ca+Mg and altitude for a dataset on monitored rivers with < 25 µg/l TP to avoid anthropogenic eutrophication (SEPA 2010) (Eqs. S2 and S3 in the supplementary material).

For rivers with more than 10% agricultural land in the catchment, results from the calculation of source apportioning of nutrient loads to the sea in the Swedish reporting to HELCOM were used (Ejhed et al. 2018). The modelled site-specific background root zone leaching, and for P also surface runoff, forms the basis for the reference value for the agricultural rivers, which is calculated as an area-weighted average of the two reference values (Eq. S4 in the supplementary material).

Sweden has no official RCs for N in fresh water, but a similar method as for P has been suggested by Fölster and Djodjic (2015) and is used in this paper. This method involves a regression model for non-agricultural land that was developed from total carbon and nitrogen deposition values (Eq. S5 in the supplementary material).

## Nutrient RCs in Nordic GIG river types in Finland, Norway and Sweden

**Fig. 3:**
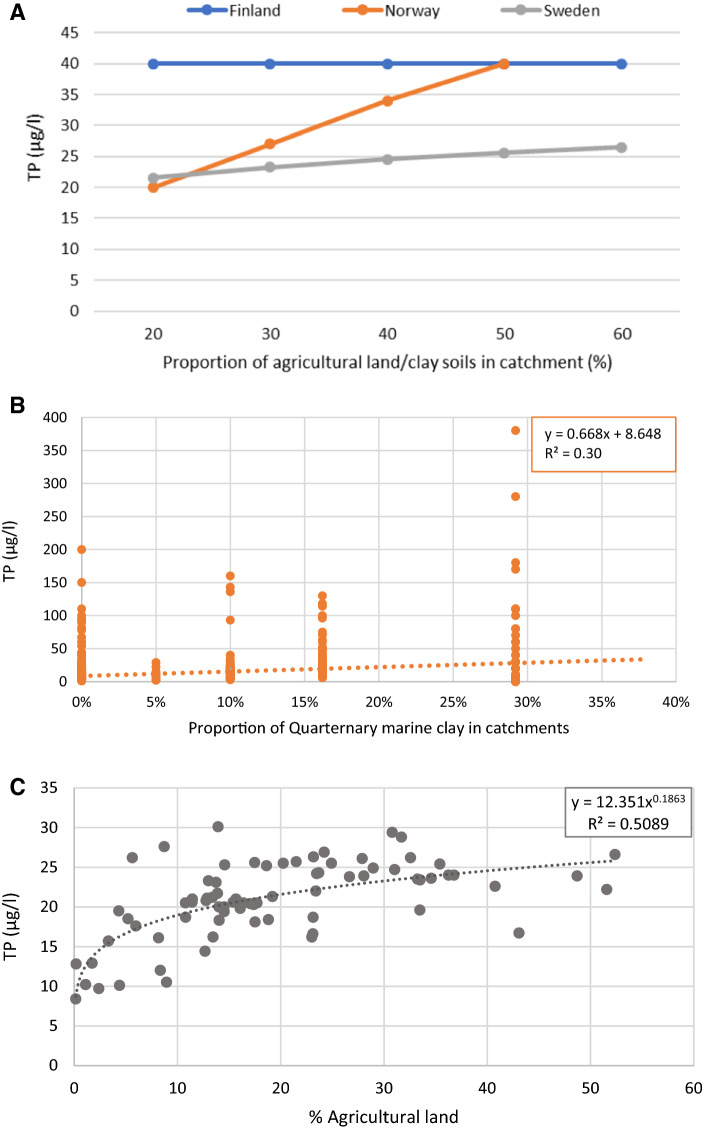
Comparison of RCs for TP in Finland, Norway and Sweden (**a**); the Norwegian (**b**) and the Swedish (**c**) data basis for setting RCs in clay-rich soils and agricultural lands, respectively. Note that the Finnish value in (**a**) represents the upper value in RC nutrient range (High/Good status class boundary)

For the five Nordic GIG river types, both TP and TN RC levels were relatively similar in the three countries, considering that the Finnish values represent the High/Good boundaries for water quality (Table 2). Higher deviations between the countries were found for the rivers draining clay-rich soils (as shown in Fig. 3a). Finland has set the High/Good boundary for TP at <40 µg/l, while the RCs of Norway and Sweden are based on regression analyses of TP concentrations and the catchments’ coverage of, respectively, clay-rich soils (NO) and agricultural land (SE). Figure 3b shows the data from five smaller forested streams that constitute the Norwegian basis for the linear regression (R^2^: 0.3), whereas Fig. 3c shows the Swedish data from streams in tile-drained fallow for which a power equation regression gave the best correlation (R^2^: 0.5). As shown, the correlations are poor, especially for the Norwegian data set where also the number of rivers is few, thereby adding to the uncertainty. An attempt to increase the number of rivers was done in 2014-2016, but the monitored streams had very steep slopes, which gave very high suspended sediment and total phosphorus concentrations (Skarbøvik, unpublished material). Hence, these data were not used in the revision of the national classification guidelines in 2018 (Direktoratsgruppen 2018).

**Table 2:** RCs of TP and TN (µg/l) for five common Nordic river types defined by the Northern GIG, and rivers draining clay-rich soils. n.d.: Not determined

Type	River characteristics	Range of TP and TN reference levels (µg/l)					
		Norway		Finland^a^		Sweden	
		TP	TN	TP	TN	TP	TN^b^
R-N1	Small lowland siliceous, moderate alkalinity	9 (1–15)	275 (1–425)	< 15	< 335	10 (6–14)^c^	306 (153–870)^c^
R-N3	Small/medium lowland organic	9 (1–17)	275 (1–425)	< 20	< 450	12 (9–19)	424 (309–692)
R-N4	Medium lowland siliceous, moderate alkalinity	9 (1–15)	275 (1–425)	< 15	< 335	9 (5–14) ^c^	337 (136–573)^c^
R-N5	Small mid-altitude siliceous	5 (1–8)	150 (1–250)	< 15	< 335	4 (3–6)	147 (100–210)
R-N9	Small/medium mid-altitude silicious, low alkalinity, organic (humic)	8 (1–13)	250(1–400)	< 20	< 450	9 (5–12)	341 (170–557)
Clay rivers	Lowland Clay-rich	20–40^d^	325	< 40	n.d.	8–30	290–775

^a^For Finland, the values represent the upper value in nutrient concentration range in RCs (High/Good status class boundary)

^b^Non-official data based on a suggestion from Fölster and Djodjic (2015)

^c^R-N1 and R-N4 in Sweden: No clear-water rivers found within those types in the dataset. Reference values calculated from corresponding humic types with colour set to a random value with the same distribution of other clear types with data

^d^Depending on marine clay coverage, see text for explanations

## Nutrient RCs in Central/Baltic GIG lowland river types in Denmark and Sweden

Table 3 shows the nationally agreed RCs for TP in the Central/Baltic GIG river types in Sweden, the values for TN being calculations based on Fölster and Djodjic (2015). The Danish RCs for nutrients were estimated based on the three methods outlined in the Methods section (direct monitoring, the Swedish model and the Norwegian regression equation for clay-rich catchments). The results revealed the following:

**Table 3:** Average concentration levels of Total P and Total N monitored in lowland Danish streams (± SD), and modelled concentrations using Swedish models for total N and P RC, as well as concentrations with minimal agricultural impacts, for the two broad Central /Baltic GIG types R-C1 and R-C6; and rivers with high proportion of clay soils assessed with the Norwegian method. n.d.: Not determined

Type	River characteristics	Denmark using monitoring of least disturbed catchments (LDC)^a^			Denmark by Norwegian method	Denmark by Swedish method		Sweden	
		No. of stations	TP	TN	TP	TP^b^	TN^b^	TP	TN^c^
R-C1	Small lowland siliceous sand^d^	6	µg/L						
			79 ± 43	805 ± 384		14–18	374–2015	21 (11–29)	428 (272–653)
R-C6	Small lowland calcareous^d^	8	5**7** ± 32	1202 ± 404		12–14	313–1095	22 (16–30)	384 (273–527)
Clay rivers	Lowland; Clay-rich	–	n.d.	n.d.	10–30^e^	12–32	244–693	8–30	290–775

^a^Since no official reference values exist for Danish rivers, the given concentrations in this table derive from the total of 16 monitored catchments in 2011 of which the 14 could be placed in one of the types and correspond to Stoddard et al. (2006)’s least disturbed conditions (LDC)

^b^The low concentration value is the true reference concentration and the high value is when allowing for a minimal pressure from low intensity agriculture in the catchment; both values are calculated with the Swedish model (Eqs. S3 and S4 for TP and S5 for TN)

^c^Non-official data based on a suggestion from Fölster and Djodjic (2015)

^d^Information on substrate and width should be known for this typology, but is not

^e^The span is explained by either using 41% clay coverage (including lower soil layers) or just 10% coverage (only including the surface layers)

**Fig. 4:**
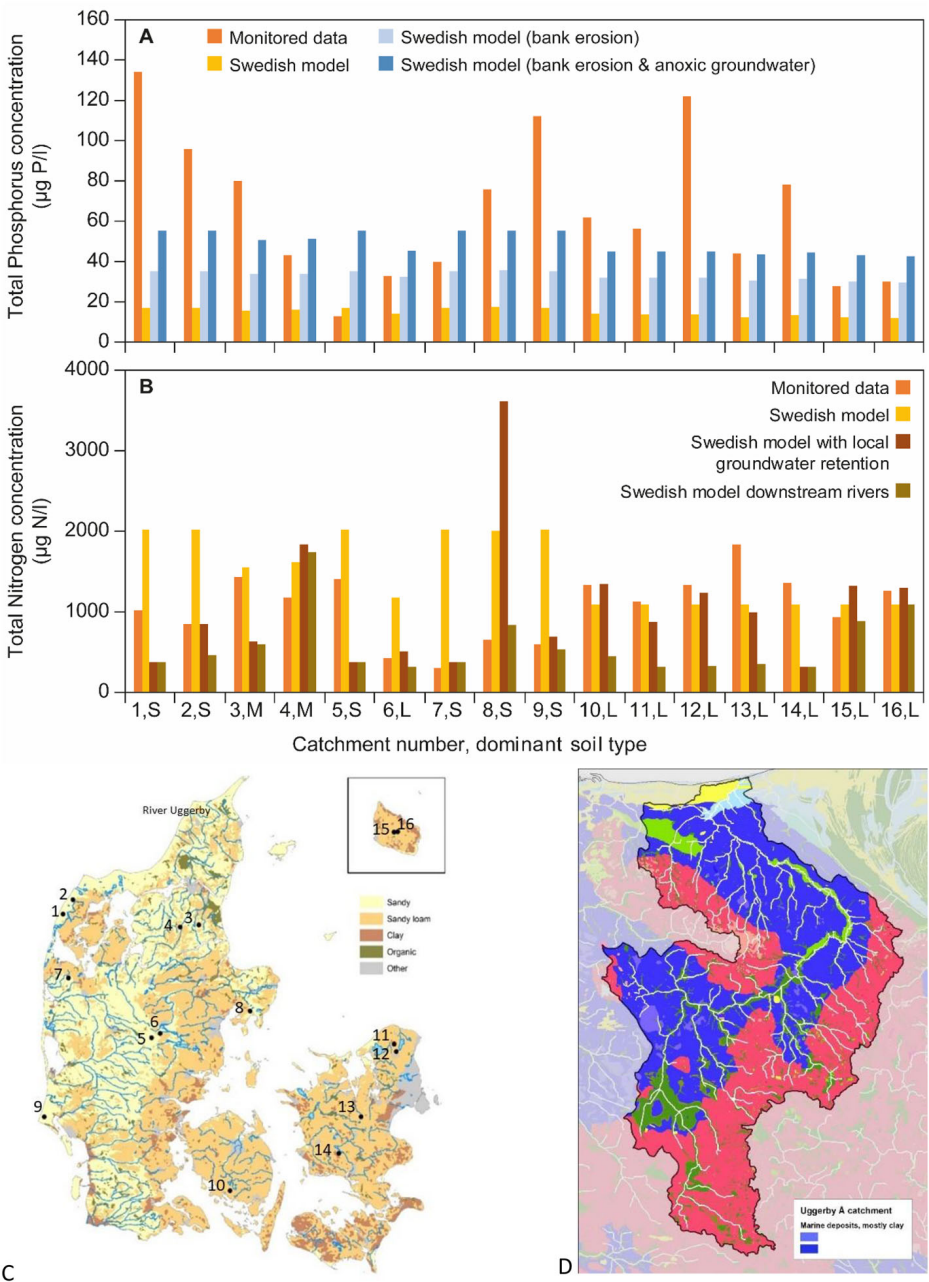
Annual average TP (**a**) and TN concentrations (**b**) from the monitored 16 smaller 1st order streams draining natural catchments with less than 10% agriculture (stations shown in the soil map (**c**) and the catchment of River Uggerby with marine deposits in D. See text for explanations of the four bars. Dominant soil types: S: Sandy soils, corresponding to Small lowland siliceous sand in Table 3. L: Sandy loam, corresponding to Small lowland calcareous in Table 3. M: Mixed soils (not used in the calculation of RCs in Table 3)

The actual data from the 16 Danish streams in least disturbed conditions had considerably higher TP concentrations than when using the Swedish model (Fig. 4a; 1st and 2nd bars, respectively). The Swedish method accounts for leaching of dissolved P but excludes input of particulate P caused by natural bank erosion. However, as bank erosion as a P source is important in lowland Danish streams (Kronvang et al. 2012), this was accounted for by adding the concentration of particulate P (average: 18 µg P/l) in the 16 catchments to the original output from the Swedish model. The resulting TP concentrations (Fig. 4a, 3rd bar) were closer to the monitoring results, but large differences remained for several streams. This is especially true for streams located in the Jutland draining windblown sandy areas showing a high proportion of particulate P (streams No. 1, 2 & 9 in Fig. 4), and one stream on Zealand draining peat areas and having high proportion of dissolved P (stream No. 12 in Fig. 4) (supplementary material). It should also be considered that other sources of nutrients may occur in the monitored catchments, such as sewage from cottages and scattered dwellings. In the last step, RCs further downstream the Danish river systems were estimated by adding modelled inputs of dissolved P from anoxic groundwater (Kronvang et al. 2007) (Fig. 4a; 4th bar).

For TN, the monitoring results gave lower RCs than the Swedish model for sandy catchments, whereas the monitoring and model results were more similar for loamy catchments (Fig. 4b; 1st and 2nd bars, respectively). The Danish sandy catchments often have a primary groundwater aquifer where substantial denitrification can take place below the redox zone (Højbjerg et al. 2015), and the standard retention factor in the Swedish model may therefore be too low (supplementary material). More comparable RCs for TN were obtained when the Swedish modelled N retention was substituted with the locally estimated N retention for the Danish catchments (Fig. 4b; 3rd bar). However, a major deviation was found for one stream (No. 8,S), probably because its catchment has a very low model-estimated N retention factor (0.09) (supplementary material). In the last step, N retention in surface waters (rivers and lakes) further downstream towards the coast was included to represent the total N concentration in rivers when they enter coastal waters (Fig. 4b). TN concentrations in the downstream rivers were in most cases considerably lower than in upstream 1st order streams (Fig. 4b; 4th bar).

The Norwegian regression between TP and the proportion of marine clay in the catchments (Eq. S1 in the supplementary material) was tested for River Uggerby (348 km^2^) situated in a clay-rich catchment in northern Denmark since none of the 16 small catchments were located on clay soils. River Uggerby has a high average suspended sediment concentration of 20–30 mg/l (Thodsen et al. 2019), suggesting that bank erosion may be considerable. Quaternary marine clay covers 41% of the catchment, but since there are aeolian deposits on top, less than 10% of the clay is found in the surface layers. This gave rise to the two assumptions that either the river bed had eroded into most of the marine clay layers (=41% coverage), yielding an RC of 30 µg/l TP, or the river was only affected by the surface soils (as presumed in Norway), yielding an RC of 10 µg/l TP. This illustrates the fact that differences in geology complicate a direct transfer of the Norwegian method to other countries.

## Discussion

The Nordic countries have chosen different methods to establish nutrient RCs in rivers, and whereas some of these methods are transferable to neighbour countries (modelling), others are not (expert judgement). The RCs for similar water types did not in general differ greatly between the countries, but the uncertainty is high, especially for lowland rivers where the catchments are heavily impacted by agriculture and settlements. The uncertainty has resulted in a relatively wide range in RCs, and this will multiply if the RCs are used to find the G/M boundaries.

In the lowland regions, monitored data give but limited information, and must be combined with other methods, such as models, historical records, expert judgement or combinations of these. Several authors have noted that the choice of method can have significant impacts on the levels of nutrient thresholds. In a comparison of nutrient criteria between the EU member states, Poikane et al. (2019) found that expert judgement-based methods resulted in less stringent G/M boundaries than data-driven approaches. Moreover, in a Canadian study of different nutrient target methods, empirical and modelling approaches provided less stringent results than ecological approaches (Chambers et al. 2008). Hawkins et al. (2010) reviewed over 1000 papers on this matter and observed that site-specific modelling was increasingly adopted due to their usually more accurate and precise determinations than the coarser estimates based on typology groups.

Of the four Nordic countries studied here, Sweden is the only one that has developed a site-specific model. Similar models, using altitude and alkalinity, have been developed in, for instance, the UK (UKTAG 2013) where RCs for TP in lowland rivers with low and high alkalinity were estimated to 19 µg/l and 36 µg/l, respectively (Defra 2014). Interestingly, new knowledge of the relationship between P concentrations and the response of river plant communities has led to a revision with significantly lower RCs than those in the former 2009-guidance (UKTAG 2013, Defra 2014). This points to the importance of repeated evaluations of methods and RC levels based on new data on nutrient-sensitive biological quality elements. Empirical models have also been developed in the US (Smith et al. 2003) based on data from 63 minimally impacted basins describing the background yield of nutrients as functions of annual runoff, basin size, the atmospheric nitrogen deposition rate and region-specific factors such as geology and soil type. The authors suggested three classes of RCs for TP: 0-30, 30-60 and >60 µg/l, and for TN: 0-150, 150-300 and >300 µg/l.

**Fig. 5:**
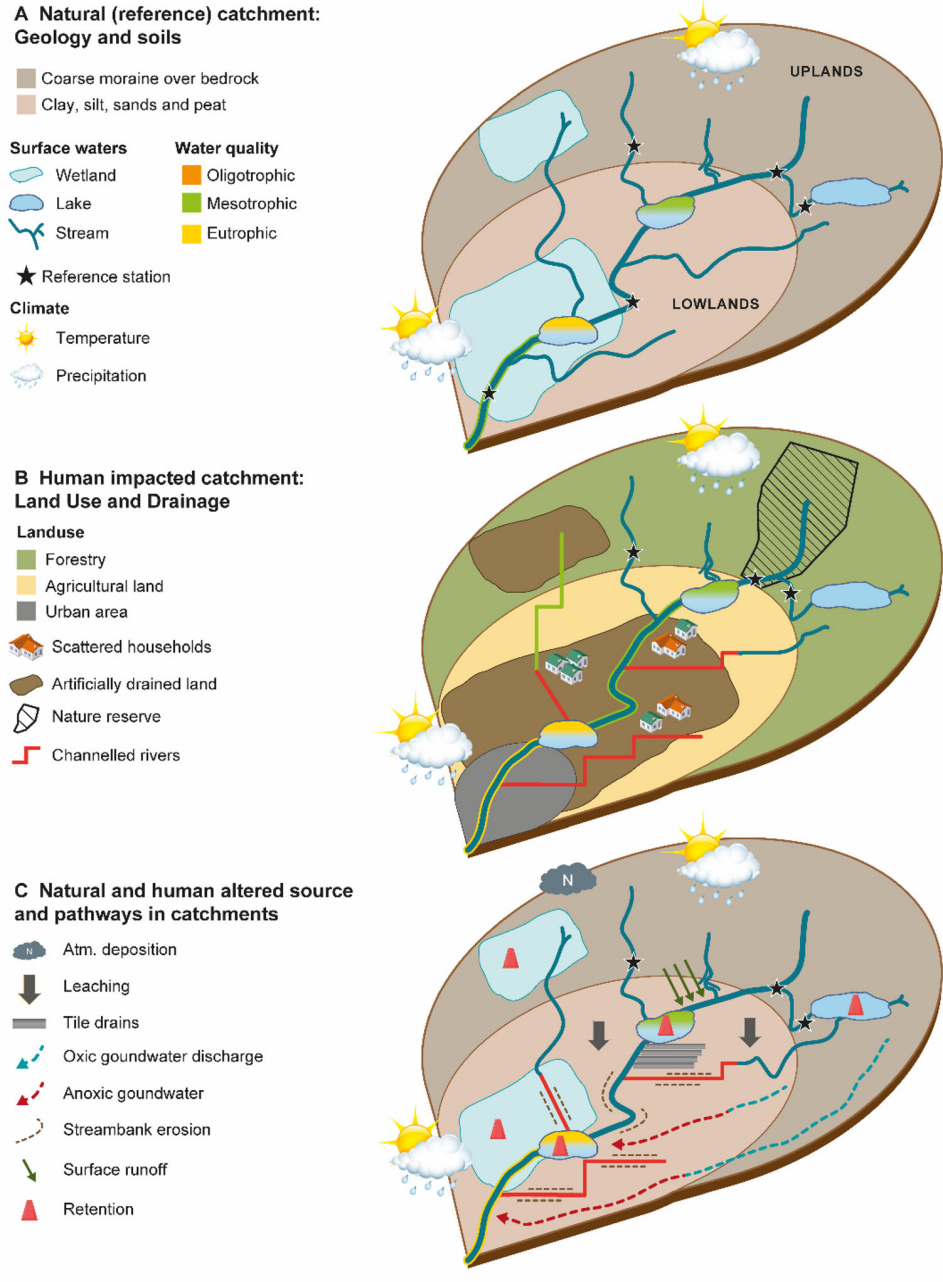
Natural reference catchment (**a**) and catchment impacted by anthropogenic modifications (**b**), illustrating the challenge of finding pristine rivers in lowland areas; and sources and pathways of nutrients in both natural and impacted catchments (**c**)

Models have the advantage that they may be used across borders. However, testing the Swedish model on Danish streams made it clear that the model was adapted to the specific climatic variations, soils and natural catchment processes in Sweden, and modifications are therefore needed before its possible transfer to neighbouring countries. Among others, we suggest inclusion of natural bank erosion, a process that may result in high inputs of P-rich soils to rivers (Kronvang et al. 2012; Skarbøvik 2016), as well as a better consideration of site-specific anoxic groundwater contributions. Moreover, the importance of clay-rich soils should be better represented in the current Swedish model, for instance by further developing the Norwegian model to establish RCs in clay-rich rivers. Figure 5 illustrates the challenges inherent in determining RC from modelling, where Fig. 5a, b depicts pristine and impacted catchments, respectively, whereas Fig. 5c shows the nutrient sources and pathways in both natural and human-impacted catchments.

In the development of a new model, it is prudent to question if tile-drained fallow land should represent reference conditions, since more nutrients can leach from this land use type than from, for instance, forests (Ulén and Etana 2010). Certainly, a core challenge of setting RCs in the lowlands is that these areas have been cultivated for thousands of years, and the question is, therefore, what landscapes the ‘true’ RCs can be found in—forest, grassland, or agriculture? This is also an important question when using historical data to determine RCs. Denmark has not yet established nutrient RCs for rivers but the use of historical data from 1900 is discussed. However, at that time agriculture was widespread, 67% of the area being cropped, 8% being bare fallow and 22% being tile drained (Jensen et al. 2017). Other attempts to use historical data to determine nutrient concentrations in lowland European rivers have shown wide variations in ranges, with concentrations of TP varying between 9 and 56 µg/l and TN between 210 and 1316 µg/l (Hirt et al. 2014). Furthermore, an exercise using the model MONERIS to establish nutrient concentrations in 1880 in lowland rivers discharging to the German part of the North Sea and to the Baltic Sea catchment yielded average concentrations of TP and TN of 35 µg P/l and 1500 µg N/l, respectively (Gadegast and Venohr 2015). Interestingly, the fact that most German agricultural streams and rivers have been heavily modified by channelling and other physical factors led to the decision to characterise many of these rivers as heavily modified water bodies.^1^ Hence, instead of setting RC levels they face the question of maximum ecological potential (CIS Guidance 2003b), but this entails the risk of allowing too liberal nutrient boundaries, potentially resulting in increased eutrophication.

Setting more accurate nutrient RCs in lowland rivers is important for both ecological and economic reasons. On average, 67% of the lowland rivers in Finland, Norway and Sweden have been reported to be in less than good ecological state (Lyche Solheim et al. 2019),^2^ and in Denmark, where almost all rivers are lowland rivers, 61% are reported to be in less than good state^1^. The G/M boundaries are often linked to the RCs, and uncertainty of these values can have severe impacts—too high RCs and G/M boundaries for nutrients may result in enhanced eutrophication and risks of harmful algal blooms (Carvalho et al. 2013), whereas too strict RCs and G/M boundaries may cause implementation of unnecessary nutrient reduction measures with significant economic consequences (Davis et al. 2015, Phillips et al. 2018). Less stringent RCs and G/M boundaries also entail the risk that managers may more readily allow new activities in a catchment such as intensified forestry or agriculture. For reasons such as these, various authors have questioned if the process of estimating RCs is rather policy-driven than based on science (Moss 2008, Bouleau and Pont 2015), supporting the argument that the methodologies for finding RCs should be transparent and harmonised. In the Nordic countries studied here, the RCs were set relatively early after the implementation of the WFD, and the uncertainty related to RCs for some lowland river types strongly points to the need for introducing more harmonised and transparent methods.

## Conclusions

The Nordic countries have chosen different methods to arrive at RCs, especially in lowland rivers where pristine conditions are rarely found. The resulting RCs are relatively comparable with some exceptions, but there is still a high degree of uncertainty in the RCs since all methods have their limitations. Establishment of RCs is a useful benchmark for assessing changes in rivers caused by land use and climate change. Therefore, RCs are becoming increasingly important, not least due to the expected land use changes in rural regions with the shift to bioeconomy. Except for Denmark, where the work on defining reference conditions is not yet finalised, establishment of reference conditions in the Nordic countries was completed in the early years after implementation of the WFD. Our findings strongly suggest the need for collection and use of new evidence data as well as development of sound models that incorporate regional catchment characteristics and processes. Preferably, a common model should be developed, taking into consideration all relevant processes in both natural catchments and catchments modified by human activities for centuries.

## Electronic supplementary material

Below is the link to the electronic supplementary material.

Supplementary material 1 (PDF 851 kb)

## Footnotes

1 Data from WISE dashboard: (https://www.eea.europa.eu/themes/water/european-waters/water-quality-and-water-assessment/water-assessments/ecological-status-of-surface-water-bodies).

2 The proportion of clay rivers is not known.
