# Supplementary material for: Comparing nutrient reference concentrations in Nordic countries with focus on lowland rivers
Source: Ambio. 2020 Sep 15;49(11):1771–83. doi: 10.1007/s13280-020-01370-4 (PMC7502649; doi:10.1007/s13280-020-01370-4)
Supplement: Supplementary file 1 — Supplementary material 1 (PDF 851 kb) [file 13280_2020_1370_MOESM1_ESM.pdf]

Ambio

Electronic Supplementary Material

This supplementary material has not been peer reviewed

Title: **Comparing nutrient reference concentrations in Nordic countries with focus on lowland rivers**

Eva Skarbøvik, Jukka Aroviita, Jens Fölster, Anne Lyche Solheim, Katarina Kyllmar, Katri Rankinen and Brian Kronvang

1. Characteristics of the common broad river types for Nordic countries

Table S-1 shows the characteristics of the seven common river types of Denmark, Finland, Norway and Sweden according to the work of the GIGs (van de Bund, W. 2009); and a river type identified as common by the authors (rivers draining clay-rich soils). R: Rivers; N: Northern GIG; C: Central-Baltic GIG. Source: EC (2018). (n.a.: not applicable).

Table S-1. Characteristics of seven common river types of Denmark, Finland, Norway and Sweden according to the work of the GIGs (van de Bund 2009); and a river type identified as common by the authors (rivers draining clay-rich soils). R: Rivers; N: Northern GIG; C: Central-Baltic GIG. Source: EC, 2018. (n.a.: not applicable).

**Type**	**River characteristics**	**Catchment area**	**Altitude, geomorphology**	**Alkalinity (meq/l)**	**Organic material (mg Pt/l)**	**Nordic Country**
**R-N1**	Small lowland siliceous, moderate alkalinity	10-100 km^2	<200 m or below the highest coastline	0.2-1	<30	FI, NO, SE
**R-N3**	Small/medium lowland organic, low alkalinity	10-1000 km^2	<200 m or below the highest coastline	<0.2	>30	FI, NO, SE
**R-N4**	Medium lowland siliceous, moderate alkalinity	100-1000 km^2	<200 m or below the highest coastline	0.2-1	<30	FI, NO, SE
**R-N5**	Small mid-altitude siliceous, low alkalinity	10 -100 km^2	Between lowland and highland	<0.2	<30	FI, NO, SE
**RN-9**	Small/medium mid-altitude siliceous low alkalinity organic (humic)	10-1000 km^2	Between lowland and highland	<0.2	>30	FI, NO, SE
**R-C1**	Small lowland siliceous sand*	10-100 km^2	Lowland, dominated by sandy substrate (small particle size), 3-8 m width (bankfull size)	>0.4	<30	DK, SE
**R-C6**	Small lowland calcareous*	10-300 km^2	Lowland gravel substrate (limestone), width 3-10 m (bankfull size)	>2	-	DK, SE
**Clay river**	Lowland, high clay content**	No known size limit	Lowland Clay soils	n.a.	n.a.	DK, FI, NO, SE

* Denmark also includes streams having a smaller catchment area than 10 km^2 in their typology.

** See country-specific definitions below.

The official definitions of clay rivers vary between the countries, as follows:

- Finland: Rivers where mean or median turbidity exceeds 5 FTU during winter low flow season (Aroviita et al. 2019);
- Norway: Rivers where the annual mean SS concentration exceeds 10 mg/l, for at least 12 samples a year (Lyche Solheim et al. 2008);
- Sweden: More than 20 % clay content (on average) in the agricultural soils of the catchment. Above that level, the background nutrient concentration is elevated (Johnsson et al 2016)
- Denmark has not yet identified a definition of this river type.

2. Methods used to estimate reference conditions in rivers for TP and TN

The WFD Annex V has a normative definition for nutrients in high ecological status, which is: “Nutrient concentrations remain within the range normally associated with undisturbed conditions.” The definition for good ecological status in Annex V is: “Nutrient concentrations do not exceed the levels established so as to ensure the functioning of the ecosystem and the achievement of the values specified above for the biological quality elements.” So, the requirements for setting reference conditions representing high status are not directly linked to the biology, while the nutrient concentrations representing good status must be linked to the biology.

Finland

In Finland, the reference conditions for TP and TN concentrations were established for the assessment of ecological quality for the first WFD river basin management planning cycle in 2008 (Vuori et al. 2009). At that time, the reference conditions and class boundaries set for nutrients were regarded preliminary but have since then been used in national status classification and river basin management (e.g. Aroviita et al. 2019). The nutrient reference values were estimated utilising monitoring data from available minimally disturbed rivers across Finland for the period 1975-2006 (Vuori et al. 2009). Annual values were used because of limited seasonal data. The river types were merged into four broad groups: small and larger rivers draining basins dominated by peatlands and small and larger rivers draining catchments with a siliceous geochemistry; the reason being that rivers in reference state do not occur in a representative pattern across the country and do not cover all the national types. Reference values were estimated utilising annual statistics (median, minimum, maximum, percentiles) within the river type groups. Reference values for rivers draining clay catchments were benchmarked according to expert judgement based on observations from minimally disturbed rivers of other types and disturbed rivers draining clay catchments. A national review panel evaluated the boundaries. Based on the annual statistics, review panel work and tests with preliminary classification results, river type-specific “high”/”good” status class boundaries for TP and TN were set as 75th-90th percentiles of the nutrient concentrations among the reference or least disturbed rivers. These boundaries equal the upper limit for nutrient concentrations in national reference conditions.

Norway

Reference conditions for total phosphorus in rivers in Norway vary from 3-11 µg/l depending on river type but are higher than this for rivers draining clay-rich soils (20-40 µg/l, depending on the proportion of clay-rich soils in the catchment) (Direktoratsgruppen 2018).

In Norway, the reference values for TP in rivers were estimated by multiplying TP values in reference lakes with comparable geochemistry by a factor 1.5 to account for a mean lake phosphorus-retention of 33% (which is close to the median of 40% reported by Brett and Benjamin, 2008).

The same principle was initially used for setting of the good/moderate status class boundaries in rivers, which vary from 8-29 µg/l for different river types, but higher for clay-affected rivers (40-80 µg/l depending on the proportion of clay in the catchment). The boundaries predicted in this simple way were later found to correspond surprisingly well with those derived from BQE-Total-P regressions using the intercalibrated indices for phytobenthos (Schneider and Lindstrøm 2011) and for benthic fauna (data from Norwegian low-alkalinity rivers compiled by T. Bækken, regression done by T.E. Eriksen and J. Moe, NIVA, see Figure 6.2 in Phillips et al. 2019). These nutrient boundaries have recently been validated using the tool kit developed for the European Best Practice Guide for establishing nutrient concentrations to support good ecological status (Phillips et al. 2019) and were found to be valid in terms of being in line with good status for the nutrient sensitive biological elements.

The ratio between the nutrients reference values and the good/moderate boundaries in Norwegian rivers is 0.33-0.42 depending on river type, except for rivers draining clay-rich soils, where the EQR-ratio is 0.5 (Direktoratsgruppen 2018).

The reference conditions for TN in rivers were also based on TN concentrations in lakes. Since Nordic reference lakes are normally phosphorus limited, they have little retention of nitrogen. Therefore, the reference levels of TN were assumed to be similar for rivers and lakes.

The reference values for TP and TN were included in the last version of the official Norwegian classification guidance for ecological status (Direktoratsgruppen 2018) and are currently used to classify ecological status in river water bodies based on monitoring data. If the biology is found to be in moderate or worse status, then the nutrients are not used in the classification, but if the biology is high or good, then also the nutrients are used for classification following the one-out-all-out-principle of downgrading the overall ecological status to moderate status.

For Norwegian rivers draining clay-rich soils, a considerable part of the particulate phosphorus derives from the phosphorus in the clay-mineral apatite. Hence, the correlation between suspended solids (SS) and TP is therefore usually good in clay-rich rivers (e.g. Skarbøvik and Roseth 2014). Thus,

the hypothesis was forwarded that the higher the proportion of clay soils in a catchment, the more clay can be eroded, and the higher the SS and TP concentrations. A total of seven streams draining clay soils in catchments mainly covered by forest were used to produce a simple linear regression between TP_{ref} (annual mean TP concentration in µg/l in near-pristine clay-soil catchments) and the proportion of the catchment area covered by marine clay (MC) (Lyche-Solheim et al. 2008):

$$TP_{ref} = 8.65 + 0.67 \bullet MC \qquad \text{(eq. S-1)}$$

MC (%) was simply found by using superficial deposit maps without considering the depth of the clay deposits. Supposing 100 % clay coverage, the maximum reference TP concentration would then theoretically be 75 µg/l, but as no rivers >10 km^2 in Norway have such high clay coverage, the maximum reference condition for TP was set to 50 µg/l (Direktoratsgruppen, 2018).

Some major challenges of this model were that R^2 was rather low when all the data were used (0.3) (although the correlation was better when using the arithmetic mean for the rivers; R^2=0.88); furthermore, most of the catchments had an MC less than 20 %, and only one catchment had an MC of 30 %.

Sweden

Sweden has used predictive modelling for setting reference conditions of TP in rivers since 1990. In the latest revision of reference conditions in rivers made in 2007, the model consisted of a linear regression of TP as a function of water colour value, non-marine Ca+Mg (calcium + magnesium), and altitude for a dataset on monitored rivers with < 25 µg/l TP to avoid anthropogenic eutrophication (Wilander 2004; Naturvårdsverket 2007). Due to this site-specific modelling, no ready-made tables exist of reference conditions for different water types in Sweden. Hence, for this paper, Swedish model outputs were calculated for the selected water types to allow comparisons with the other countries.

Sweden is dominated by forests and mires located mainly on granitoide till soils, and for rivers draining these soil types, the following equation was developed:

$$^{10}\log P\text{-ref} = 1.533 + (0.240 \bullet {}^{10}\log(Ca^{*}Mg^{*})) + (0.301 \bullet {}^{10}\log(AbsF)) - 0.012\,(Altitude)^{0.5} \qquad \text{(eq S-2)}$$

where P-ref is the reference value (TP µg/l), Ca*Mg* is non-marine base cations (meq/l), calculated as Ca + Mg – 0.235•Cl, AbsF is absorbance measured at 420 nm in 5 cm cuvettes[1], and altitude is the height of the sampling station (m asl).

This regression model is used for rivers with less than 10% agricultural land in the catchment area. If data on non-marine base cations are not available, a simplified model is used[2]:

$$^{10}\log P\text{-ref}=1.380+(0.240\bullet{}^{10}\log(AbsF))-(0.0143\bullet(Altitude)^{0.5}) \qquad \text{(eq S-3)}$$

For rivers draining agricultural land, a different equation was developed using results from the calculation of source apportioning of nutrient loads to the sea in the Swedish reporting to HELCOM (Ejhed et al. 2018). Root zone leaching of TP from unfertilized fallow was modelled by using the ICECREAM model (Ejhed et al. 2016; Larsson et al. 2007) and applied as an estimate of background leaching (Larsson et al. 2007). Important driving variables for the model are climate, soil type, soil P content and slope. Half of the leached TP was assumed to be retained in the soil or in ditches, and the reference values were therefore set to half the concentration of the modelled background leaching. For water bodies with mixed land use in the catchment, holding more than 10% agricultural land, the reference value is calculated as an area-weighted average of the two different models according to the following equation:

$$Pagr\text{-}ref = ((Pagr \bullet Aagr \bullet 0.5) + (P\text{-}ref\bullet(100\text{-}Aagr))/100 \qquad \text{(eq S-4)}$$

where $Pagr_{ref}$ is the co-weighted reference value (TP µg/l) in areas with agricultural land; Pagr is the modelled reference value (TP µg/l) for agricultural land in the specific area (ICECREAM model); Aagr is the percentage (%) of agricultural soil in the area; P-ref is the reference value for “non-agricultural soil” in accordance with the above; and 0.5 is a specific factor for weighting in the status classification, reflecting the retention of TP between the root zone and the stream.

This reference value reflects a state with no fertilization or ploughing but with tile drainage of most clay soils. The background leaching of particulate phosphorus is then regarded as mainly originating from internal erosion in soil cracks and the tile drainage system. Since the rivers are slow flowing, bank erosion is regarded negligible as compared with erosion from the fields.

---

[1] To convert to mg Pt/l, multiply by 500.
[2] This is the model used by the other Nordic countries in the discussion part of this paper.

Sweden has not defined reference conditions for nitrogen in freshwater since phosphorus is regarded as the limiting factor for eutrophication in freshwaters. However, in our paper, we have used a classification of total nitrogen following the same principles as for phosphorus with a regression function, and modelled background leaching from the reporting to HELCOM for agricultural soils (Fölster and Djodjic 2015). The regression equation for the reference value of total nitrogen (TM_{ref}) includes the parameters total organic carbon (TOC) and nitrogen deposition at the year 2008 ($Ndep_{2008}$):

$$logTN_{ref} = 1{,}597 + 0{,}614 * logTOC + 0{,}147 * LogNDep_{2008} \qquad \text{(eq S-5)}$$

The equation reflects that most of the background nitrogen is organic bound to humic substances. The inclusion of NDep as explaining variable means that the elevated nitrogen leaching from forests and wetlands due to nitrogen deposition the last century is neglected. The elevated leaching from deposition does however seldom include inorganic reactive nitrogen, but rather organic matter with an elevated nitrogen content.

For the root zone leaching of nitrogen from agricultural soils, the SOILNDB model was used (Johnsson et al. 2002), and for mixed land use, the corresponding formula for area weighing as for Pref was used (eq S-4).

Denmark

Since reference conditions have not yet been finally determined for Danish rivers, we made use of the preliminary work conducted within the framework of the first and second river basin management plans (RBMPs) under the WFD. Data from 19 catchments throughout Denmark, with less than 10 % agricultural land, were used to determine TN and TP levels under least disturbed conditions. The rivers were screened for nutrient concentrations and discharge in 2004/2005 (Kronvang et al., 2015). Presently, 16 of the 19 rivers are included in the Danish National Aquatic Monitoring Programme (NOVANA) and have been monitored every third year since 2011 (Thodsen et al. 2019); see Table S-2. In the paper, we explain how these 16 streams were used for a comparison between the monitored data and the Swedish method.

Table S-2. Overview of the 16 streams monitored for assessing the Least Disturbed Conditions in nutrient concentrations under the Danish national environment and nature monitoring programme (NOVANA), with catchment area, the model calculated nitrogen retention in groundwater and surface waters, as well as the potential average modelled concentration of dissolved phosphorus discharging to river channels in Denmark.

Stream Number	Station ID-number Danish monitoring programme (NOVANA)	Catchment area (km^2)	Nitrogen retention in groundwater (%)	Nitrogen retention in surface water to downstream coastal water (%)	Phosphorus discharge with anoxic groundwater ($\mu g\ P\ l^{-1}$)
1	1000069	3.9	97	76	20
2	11000272	13.0	80	58	20
3	14000020	1.7	81	10	17
4	14000284	0.4	43	4	17
5	21000110	4.6	97	66	20
6	21000861	0.5	80	79	13
7	22000047	5.4	94	80	20
8	23000173	0.6	9	77	20
9	30000013	15.7	84	26	20
10	45000059	0.4	38	26	13
11	48000006	6.1	60	95	13
12	49000146	3.1	43	77	13
13	52000428	1.6	55	70	13
14	57000178	0.5	90	37	13
15	66000034	5.2	39	37	13
16	67000030	4.6	40	17	13

4. Proportion of lowland rivers at risk

In the discussion of the paper we have indicated the importance of Nordic lowland rivers in terms of the proportion of these that are at risk of not achieving the targets of the WFD. More details on this issue are provided here:

The total number of classified water bodies in different broad types of lowland rivers in the Nordic countries (excl. DK[3]) is 11699, based on official data reported by the countries to WISE with the 2nd RBMPs, and on links between national types and broad types (Lyche Solheim et al. 2019). These may or may not include rivers draining clay-rich soils. The proportion of these river water bodies that have been reported to be in less than good ecological status is 67%, but varying from 32% in Finland, 53% in Norway to 83% in Sweden (Table S-3).

[3] Danish rivers were not included because the Danish river typology was not based on geochemistry as type descriptor and could therefore not be linked to the broad types, although Denmark has rivers/streams belonging to the CB-GIG types R-C1 and R-C6.

The reason for the differences between the countries can be several, e.g. different pressures (type of pressure and pressure intensity), different combination rules across quality elements (whether the country has chosen to use the “one-out-all-out” principle, or other principles) or different good/moderate class boundaries. The boundaries reported to WISE show that Norway has considerably tighter G/M boundaries than Finland (Kelly et al. 2019). The Swedish boundaries could not be extracted for comparison due to reporting of EQR-values rather than concentrations. Denmark did not report any boundaries.

Table S-3. Total number of lowland riverine water bodies (tot), and the proportion of these that are in less than Good ecological status (%<G) for each broad type, as well as the corresponding intercalibration type for the Northern (NGIG) and the Central-Baltic GIG (CB-GIG) in Finland (FI), Norway (NO) and Sweden (SE).

Broad Type Code	Broad Type	Corresponding IC type (N-GIG & CB-GIG)	FI		SE		NO	
			tot	%<G	tot	%<G	tot	%<G
RW-01-01	Very large rivers	R-L1 (very large low alkalinity rivers), R-L2 (very large, medium to high alkalinity rivers)	22	55			24	46
RW-02-04	Lowland, calcareous or mixed, medium or large	R-C4 (Medium, lowland, mixed), R-C5 (Large, lowland, mixed)			54	93	10	60
RW-02-07	Lowland, calcareous or mixed (including organic)	None			354	92	28	86
RW-03-05	Lowland, calcareous or mixed, very small or small	R-C6 (Small, lowland, calcareous)			106	92	235	77
RW-04-02	Lowland, siliceous, medium or large	R-N4 (Medium, lowland, siliceous, moderate alkalinity)	295	33	358	90	93	81
RW-04-06b	Lowland, siliceous or organic, medium or large	R-N3 (Small/medium, lowland, organic, low alkalinity)	541	35	1474	90	47	60
RW-05-03	Lowland, siliceous, very small or small	R-N1 (Small, lowland, siliceous, moderate alkalinity)<br>R-C1 (Small lowland, siliceous sand)<br>R-C2 (Small lowland, siliceous rock)	278	34	877	82	2072	52
RW-05-06a	Lowland, organic and siliceous, very small or small	R-N3 (Small/medium, lowland, organic, low alkalinity)	368	24	3377	77	1086	47

References to the supplementary material

Aroviita J., S. Mitikka and S. Vienonen (eds.) 2019. Status classification and assessment criteria of surface waters in the third river basin management cycle. Reports of the Finnish Environment Institute 37/2019: 1–177. (in Finnish) http://hdl.handle.net/10138/306745

Brett, M. T. and M.M. Benjamin. 2008. A review and reassessment of lake phosphorus retention and the nutrient loading concept. *Freshwater Biology* 53: 194-211.

Direktoratsgruppen 2018. Guidance 02:2018. National Guidance on Classification of environmental state in water. Direktoratsgruppen for gjennomføring av vanndirektivet. 222 pp. (in Norwegian). http://www.vannportalen.no/veiledere/

EC 2018. Commission Decision (EU) 2018/229 establishing, pursuant to Directive 2000/60/EC of the European Parliament and of the Council, the values of the Member State monitoring system classifications as a result of the intercalibration exercise and repealing Commission Decision 2013/480/EU. Official Journal of the European Union L47/1: 91 pp.

Ejhed, H., J. Tengdelius Brunell, E. Widén Nilsson, J. Hytteborn, H. Johnsson and K. Blombäck. 2018. PM method description of calculation of PLC6 pressure on new water body classification 2016-Version PLC6.5 SMED PM. (in Swedish).

Ejhed, H., E. Widén-Nilsson, J.T. Brunell and J. Hytteborn. 2016. Nutrient pressure to the Baltic Sea and Västerhavet 2014 – Swedish contribution to HELCOM's sixth Pollution Load Compilation. Havs- och vattenmyndighetens Rapport 2016:12. (In Swedish).

Fölster, J. and F. Djodjic. 2015. Basis for assessing nitrogen in lakes and rivers. SLU, Vatten och miljö: Rapport 2015:12. (In Swedish).

Johnsson, H., Larsson, M., Mårtensson, K. & Hoffmann, M., 2002. SOILNDB: A decision support tool for assessing nitrogen leaching losses from arable land. *Environmental Modelling & Software* 17:505-517.

Johnsson, H., K. Mårtensson, A. Lindsjö, K. Persson, Y. Andrist Rangel and K. Blombäck. 2016. Leakage of nutrients from Swedish agruicultural fields – Calculations of normal leakage of nitrogen and phosphorus in 2013. SMED Rapport Nr 189. (In Swedish).

Kelly, M., G. Phillips, H. Teixeria, F. Salas-Herrero, A. Lyche Solheim, S. Poikane. 2019. Physico-chemical supporting elements: a review of national standards to support good ecological status. ECOSTAT draft report, 167 pp. https://circabc.europa.eu/w/browse/491b7b0f-bbb7-4d4f-afdc-82da0a6df90f

Kronvang, B., J. Windolf, S.E. Larsen and J. Bøgestrand. 2015. Background concentrations and loadings of nitrogen in Danish surface waters. *Acta Agriculturae Scandinavica, Section B - Soil & Plant Science* 65: 155-163.

Larsson, M. H., K. Persson, B. Ulén, A. Lindsjö and N. J. Jarvis. 2007. "A dual porosity model to quantify phosphorus losses from macroporous soils." *Ecological Modelling* 205(1): 123-134.

Lyche Solheim, A., D. Berge, T. Tjomsland, F. Kroglund, I. Tryland, A.K. Schartau, T. Hesthagen, Borch, H. et al., 2008. Suggested environmental targets for physical-chemical parameters in lakes and rivers, including clay-rich rivers and criteria for user interests. Supplement for the guidance on ecological classification. NIVA-Report 5708-2008. 79 pp. (In Norwegian).

Lyche Solheim, A., L. Globevnik, K. Austnes, P. Kristensen, J. Moe, J. Persson, G. Phillips, S. Poikane, et al. 2019. A new broad typology for rivers and lakes in Europe: Development and application for large-scale environmental assessments. *Science of the Total Environment* 697. 134043, ISSN 0048-9697, doi.org/10.1016/j.scitotenv.2019.134043.

Naturvårdsverket 2007. State, potential and quality requirements for lakes, rivers, coastal waters and transitional waters. A handbook on how quality requirements in surface water bodies can be determined and followed up. Handbok 2007:4. (In Swedish).

Phillips, G., Kelly, M., Teixeira, H., Salas, F., Free, G., Leujak, W, Pitt, J.A., Lyche Solheim, A., Várbíró, G., Poikane, S., 2018. Best practice for establishing nutrient concentrations to support good ecological status. JRC Science for Policy Report. EUR 29329 EN: 142 pp. https://ec.europa.eu/jrc/en/publication/eur-scientific-and-technical-research-reports/best-practice-establishing-nutrient-concentrations-support-good-ecological-status

Schneider, S. and E.A. Lindstrøm. 2011. The periphyton index of trophic status PIT: a new eutrophication metric based on non-diatomaceous benthic algae in Nordic rivers. *Hydrobiologia* 665: 143-155.

Thodsen, H. et al., 2019. Water courses 2017. NOVANA. Aarhus University, DCE – Danish Centre for Environment and Energy, Scientific Report No. 306: 74 pp (in Danish).
http://dce2.au.dk/pub/SR306.pdf

Van de Bund, W. 2009. Water Framework Directive Intercalibration Technical Report. Part 1: Rivers. European Commission report EUR 23838 EN/1 – Joint Research Centre – Institute for Environment and Sustainability, DOI 10.2788/23384: 136 pp.

Vuori, K.-M., S. Mitikka, and H. Vuoristo (eds.) 2009. Guidance on ecological classification of surface waters in Finland. Environmental Administration Guidelines 3/2009: 1-120. (in Finnish)
http://hdl.handle.net/10138/41785

Wilander, A. 2004. Suggested criteria for assessing eutrophication. Rapport 2004:19, Institutionen för vatten och miljö, SLU. (In Swedish)
